# To freeze or not to freeze

**DOI:** 10.7554/eLife.13119

**Published:** 2015-12-23

**Authors:** Debra Bangasser

**Affiliations:** Department of Psychology and Neuroscience Program, Temple University, Philadelphia, United Statesdebra.bangasser@temple.edu

**Keywords:** fear conditioning, sex differences, active responses, Rat

## Abstract

Male and female rats respond to a fearful experience in different ways, but this was not previously taken into account in research into psychiatric disorders.

**Related research article** Gruene TM, Flick K, Stefano A, Shea SD, Shansky RM. 2015. Sexually divergent expression of active and passive conditioned fear responses in rats. *eLife* **4**:11352. doi: 10.7554/eLife.11352

**Image** Female rats are more likely to ‘dart’ than male rats when they feel fear

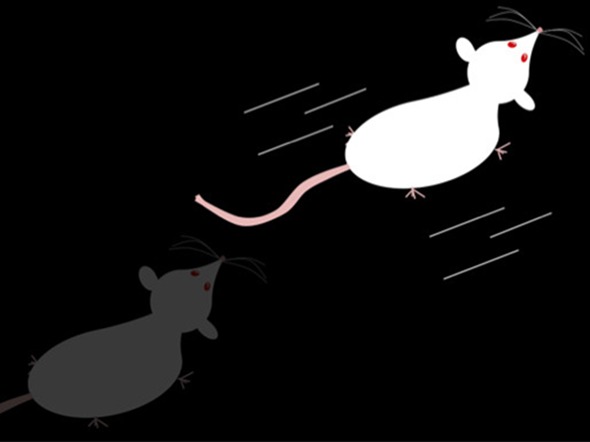

Much of our understanding of the neurobiological basis of psychiatric disorders, such as post-traumatic stress disorder and depression, is based on studies that involve rodents. However, although post-traumatic stress disorder and depression occur more frequently in women (Kessler et al., 2012), most preclinical studies in this area have been conducted on male rodents (Beery and Zucker, 2011). This means many of the tests that researchers use to measure fear, anxiety and depressive-like behavior have been validated in male rodents, but not in female rodents (Kokras and Dalla, 2014). Moreover, male and female rodents differ in terms of their size, strength and other characteristics, and these differences can influence how the animals behave. For example, female rats are more active than males, which may explain why some classic tests that define anxiety on the basis of inactivity and avoidance detect fewer behavioral changes in females than in males (Fernandes et al., 1999; Kokras and Dalla, 2014). There is, therefore, a clear need to include female rats in preclinical studies and to allow for the differences between males and females when measuring fear, anxiety and depressive-like behavior. Now, in eLife, Rebecca Shansky of Northeastern University and colleagues – including Tina Gruene as first author – report the results of experiments that found that male and female rats respond to a fearful experience in different ways (Gruene et al., 2015).

Assessing sex differences in fear responses is particularly critical because fear-conditioning tasks are widely used to study the neurobiological underpinnings of learning and remembering distressing or traumatic events. In the typical rodent fear-conditioning task, a tone that initially produces no behavioral response is paired with a slight electric shock to the foot that elicits a fear response. The tone is played so that it precedes and predicts the shock: this results in the rodent becoming ‘conditioned’ to the tone and displaying a fear response when the tone is played without the shock being applied. However, once the conditioned fear response has been learned it can be extinguished, or diminished, by playing the tone without applying the shock a number of times.

The challenge in these experiments is to find a way to measure the degree of fear. Most studies do this by measuring freezing, which is characterized by the rodents ceasing to move. Freezing is generally regarded as a valid measure of fear learning in male rodents. However, Gruene, Shansky and their colleagues – Katelyn Flick and Alexis Stefano of Northeastern, and Stephen Shea of Cold Spring Harbor Laboratories – found that instead of freezing, many female rats display a brief, high-velocity movement termed darting.

Before conditioning, the rats did not dart when they heard the tone. However, darting increased over time as the rats learned to associate the tone with a shock to the foot. Additionally, darting could be extinguished by playing the tone without applying the shock. Taken together, these findings indicate that darting, like freezing, reflects learned fear.

Gruene et al. found that female rats performed more darts per minute than males. However, not all females dart, and not all males freeze: in the experiments approximately 40% of the females engaged in darting behavior, but only about 10% of males did so. These findings reveal sex differences in the presentation and prevalence of darting behavior and illustrate that there is greater variability in the fear responses produced by female rats.

The finding that a higher proportion of female rats dart may explain why previous studies have reported less freezing in females (e.g., Maren et al., 1994; Pryce et al., 1999). Moreover, the Gruene et al. study underscores that darting behavior needs to be assessed in fear-conditioning studies in the future, particularly when females are included. Additional research should also determine the prevalence of conditioned darting in other species, such as mice, which are commonly used in fear-conditioning studies.

More broadly, when including female subjects in tests that were developed using male subjects, it is critical to observe and validate behaviors that are unique to or more commonly expressed by females. Considering these behaviors is timely because the National Institutes of Health in the US has instituted a new policy to promote the inclusion of female rodents in preclinical studies (Clayton and Collins, 2014). With more researchers studying female rodents, a failure to consider sex differences in the behavioral expression of fear, anxiety and depressive-like behavior can potentially lead to inaccurate interpretations of results, thereby hindering scientific progress. The work of Gruene et al. can serve as a model for how to validate common behavioral tests with female rodents. Finally, using behavior procedures that have been validated in both sexes will be critical to understanding the neurobiological processes that underlie post-traumatic stress disorder and depression in both men and women.
